# *N*-(2-Formyl­phen­yl)-4-methyl-*N*-[(4-methyl­phen­yl)sulfon­yl]benzene­sulfon­amide

**DOI:** 10.1107/S1600536814010666

**Published:** 2014-05-17

**Authors:** Sung-Gon Kim

**Affiliations:** a Department of Chemistry, Kyonggi University, 154-42, Gwanggyosan-ro, Yeongtong-gu, Suwon 443-760, Republic of Korea

## Abstract

In the title compound, C_21_H_19_NO_5_S_2_, the dihedral angles between the formyl­phenyl ring and the two methyl­phenyl rings are 29.3 (3) and 28.9 (3)°, respectively; the dihedral angle between the methyl­phenyl rings is 48.4 (2)°. The C—N—S—C torsion angles are −74.1 (2) and −105.4 (2)°. In the crystal, molecules are linked by pairs of C—H⋯O hydrogen bonds, forming inversion dimers.

## Related literature   

Several sulfonamide derivatives have been used as chemotherapeutic agents for their anti­bacterial, anti­fungal, anti­tumor and hypoglycemic effects, see: Chohan *et al.* (2010 ▶); El-Sayed *et al.* (2011 ▶); Seri *et al.* (2000 ▶). Some sulfonamide derivatives have been shown to possess carbonic anhydrases inhibitory properties, see: Suparan *et al.* (2000 ▶). Disulfonamides containing two sulfone groups connected to the nitro­gen atom are used for their anti­tumor activity and carbonic anhydrases inhibitory properties, see: Boriack-Sjodin *et al.* (1998 ▶). For related structures, see: Elgemeie *et al.* (2013 ▶); Mughal *et al.* (2012 ▶).
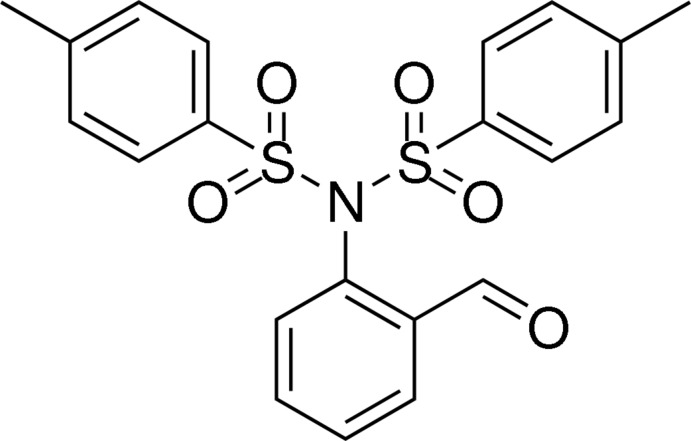


## Experimental   

### 

#### Crystal data  

C_21_H_19_NO_5_S_2_
*M* *_r_* = 429.49
Monoclinic, 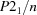

*a* = 15.5505 (6) Å
*b* = 7.8816 (3) Å
*c* = 16.6876 (7) Å
β = 103.942 (1)°
*V* = 1985.03 (14) Å^3^
*Z* = 4
Mo *K*α radiation
μ = 0.30 mm^−1^
*T* = 199 K
0.22 × 0.14 × 0.05 mm

#### Data collection  

Bruker SMART 1000 CCD area-detector diffractometer
14091 measured reflections
4943 independent reflections
3099 reflections with *I* > 2σ(*I*)
*R*_int_ = 0.045

#### Refinement  

*R*[*F* ^2^ > 2σ(*F* ^2^)] = 0.049
*wR*(*F* ^2^) = 0.173
*S* = 1.12
4943 reflections
264 parameters
H-atom parameters constrained
Δρ_max_ = 0.42 e Å^−3^
Δρ_min_ = −0.64 e Å^−3^

### 

Data collection: *SMART* (Bruker, 2007 ▶); cell refinement: *SAINT* (Bruker, 2007 ▶); data reduction: *SAINT*; program(s) used to solve structure: *SHELXTL* (Sheldrick, 2008 ▶); program(s) used to refine structure: *SHELXTL* ; molecular graphics: *ORTEP-3 for Windows* (Farrugia, 2012 ▶); software used to prepare material for publication: *SHELXTL* and *publCIF* (Westrip, 2010 ▶).

## Supplementary Material

Crystal structure: contains datablock(s) I, global. DOI: 10.1107/S1600536814010666/xu5787sup1.cif

Structure factors: contains datablock(s) I. DOI: 10.1107/S1600536814010666/xu5787Isup2.hkl

Supporting information file. DOI: 10.1107/S1600536814010666/xu5787Isup3.cml

CCDC reference: 1002056

Additional supporting information:  crystallographic information; 3D view; checkCIF report

## Figures and Tables

**Table 1:** Hydrogen-bond geometry (Å, °)

*D*—H⋯*A*	*D*—H	H⋯*A*	*D*⋯*A*	*D*—H⋯*A*
C6—H6⋯O2^i^	0.95	2.62	3.261 (4)	125

Symmetry code: (i) 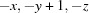.

**Fig. 1.**
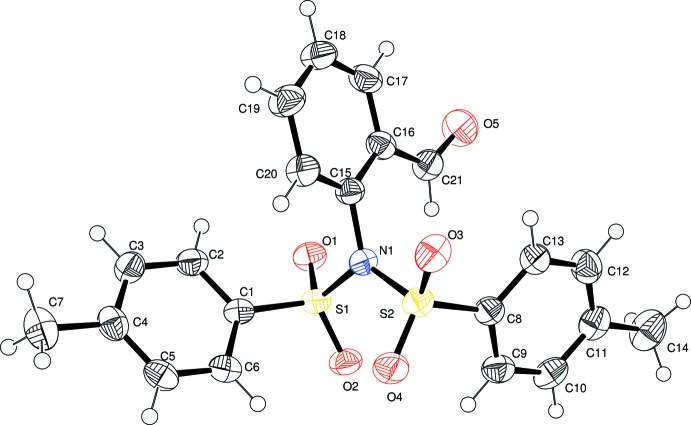
A view of the molecular structure of the title compound, showing the atom-numbering scheme. Displacement ellipsoids are drawn at the 50% probability level.

**Fig. 2.**
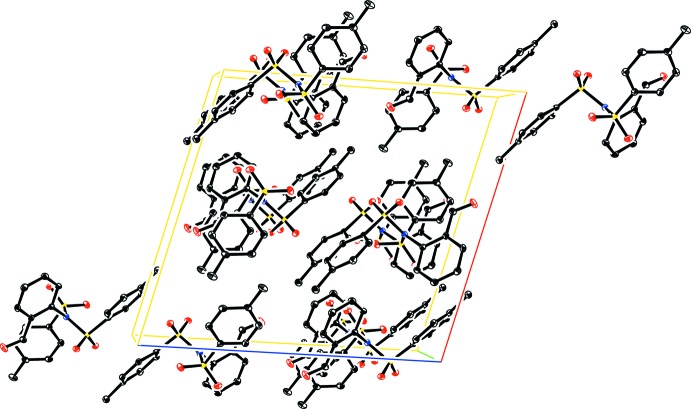
A partial view of the crystal packing of the title compound. Hydrogen atoms have been omitted for clarity.

## supplementary crystallographic information

### 2. Structural commentary

Sulfonamides, which are already known as sulfa drugs, are an important class of compounds in the field of chemistry, biology and pharmacology. Several sulfonamide derivatives are used as chemotherapeutic agents for their anti­bacterial, anti­fungal, anti­tumor and hypoglycemic (Chohan *et al.*, 2010; El-Sayed *et al.*, 2011; Seri *et al.*, 2000). In addition, some sulfonamide derivatives have been shown to inhibit on carbonic anhydrases (Suparan *et al.*, 2000). Disulfonamides containing two sulfone groups connected to the nitro­gen atom are used for their anti­tumor activity and carbonic anhydrases inhibitory properties (Boriack-Sjodin *et al.*, 1998). In view of these potential applications and in continuation of our work, the structure of the title compound has been carried out and the results are presented here.

X-ray analysis confirms the molecular structure and atom connectivity as illustrated in Fig. 1. The geometry around atoms S1 and N1 are distorted tetra­hedral and planar trigonal, respectively. The average S—O bond length is 1.421 (2) Å, while the S—N and S—C bond lengths are 1.694 (3) and 1.746 (3), respectively. The dihedral angles between the formyl­phenyl ring (C15–20) and the C1–C6 and C8–C13 methyl­phenyl rings are 29.3 (3) and 28.9 (3)^o^; the dihedral angle between the methyl­phenyl rings is 48.4 (2)^o^. The sulfonamide torsion angles are -74.1 (2)° for C15—N1—S1—C1 and -105.4 (2)° for C15—N1—S2—C8. In the crystal, Fig 2, weak C—H···O hydrogen bonds link the molecules, forming a three-dimensional network.

### 5. Synthesis and crystallization

A solution of 4 M Na_2_CO_3_ in water (35 mL) was added to a solution of 2-amino­benzyl alcohol (5.0 mmol) and *p*-toluene­sulfonyl chloride (12.0 mmol) in THF (10 mL). After stirring at room temperature for 24 h, the reaction mixture was poured into cold water and extracted with EtOAc. The resultant organic layer was washed with brine and dried over MgSO_4_. The resulting residue was purified by silica gel chromatography to afford 2-(ditoluensulfonyl­amino)­benzyl alcohol. Next, to solution of 2-(ditoluensulfonyl­amino)­benzyl alcohol in CH_2_Cl_2_ (10 mL) was added excess MnO_2_ (20 mmol). After stirring for at room temperature for 36 h, the reaction mixture was filtered under celite pad and purified by silica gel chromatography to afford the title compounds. Crystals suitable for X-ray analysis were obtained by recryatallization from an n-hexane/CH_2_Cl_2_ solution.

### 6. Refinement

All H atoms were positioned geometrically, (C—H = 0.95–0.96 Å) and constrained to ride on their parent atoms, with *U*_iso_(H) = *xU*eq(C), where *x* = 1.2 for all other H atoms.

### Crystal data

**Table:**

C_21_H_19_NO_5_S_2_	*F*(000) = 896
*M**_r_* = 429.49	*D*_x_ = 1.437 Mg m^−^^3^
Monoclinic, *P*2_1_/*n*	Mo *K*α radiation, λ = 0.71073 Å
Hall symbol: -P 2yn	Cell parameters from 5757 reflections
*a* = 15.5505 (6) Å	θ = 2.5–28.3°
*b* = 7.8816 (3) Å	µ = 0.30 mm^−^^1^
*c* = 16.6876 (7) Å	*T* = 199 K
β = 103.942 (1)°	Block, colorless
*V* = 1985.03 (14) Å^3^	0.22 × 0.14 × 0.05 mm
*Z* = 4	

### Data collection

**Table:**

Bruker SMART 1000 CCD area-detector diffractometer	3099 reflections with *I* > 2σ(*I*)
Radiation source: fine-focus sealed tube	*R*_int_ = 0.045
Graphite monochromator	θ_max_ = 28.3°, θ_min_ = 1.6°
phi and ω scans	*h* = −19→20
14091 measured reflections	*k* = −10→6
4943 independent reflections	*l* = −22→22

### Refinement

**Table:**

Refinement on *F*^2^	Primary atom site location: structure-invariant direct methods
Least-squares matrix: full	Secondary atom site location: difference Fourier map
*R*[*F*^2^ > 2σ(*F*^2^)] = 0.049	Hydrogen site location: inferred from neighbouring sites
*wR*(*F*^2^) = 0.173	H-atom parameters constrained
*S* = 1.12	*w* = 1/[σ^2^(*F*_o_^2^) + (0.0595*P*)^2^ + 2.1799*P*] where *P* = (*F*_o_^2^ + 2*F*_c_^2^)/3
4943 reflections	(Δ/σ)_max_ < 0.001
264 parameters	Δρ_max_ = 0.42 e Å^−^^3^
0 restraints	Δρ_min_ = −0.64 e Å^−^^3^

### Special details

**Table:**

Geometry. All e.s.d.'s (except the e.s.d. in the dihedral angle between two l.s. planes) are estimated using the full covariance matrix. The cell e.s.d.'s are taken into account individually in the estimation of e.s.d.'s in distances, angles and torsion angles; correlations between e.s.d.'s in cell parameters are only used when they are defined by crystal symmetry. An approximate (isotropic) treatment of cell e.s.d.'s is used for estimating e.s.d.'s involving l.s. planes.
Refinement. Refinement of *F*^2^ against ALL reflections. The weighted *R*-factor *wR* and goodness of fit *S* are based on *F*^2^, conventional *R*-factors *R* are based on *F*, with *F* set to zero for negative *F*^2^. The threshold expression of *F*^2^ > σ(*F*^2^) is used only for calculating *R*-factors(gt) *etc*. and is not relevant to the choice of reflections for refinement. *R*-factors based on *F*^2^ are statistically about twice as large as those based on *F*, and *R*-factors based on ALL data will be even larger.

### Fractional atomic coordinates and isotropic or equivalent isotropic displacement parameters (Å^2^)

**Table:**

	*x*	*y*	*z*	*U*_iso_*/*U*_eq_	
N1	0.04365 (17)	0.3510 (3)	0.23535 (16)	0.0354 (6)	
S1	−0.02134 (5)	0.27690 (10)	0.14551 (5)	0.0346 (2)	
O1	−0.07076 (14)	0.1425 (3)	0.16970 (14)	0.0414 (6)	
O2	−0.06558 (15)	0.4204 (3)	0.10297 (14)	0.0427 (6)	
S2	0.07742 (5)	0.55504 (11)	0.25037 (5)	0.0365 (2)	
O3	0.15148 (15)	0.5490 (3)	0.31970 (15)	0.0478 (6)	
O4	0.08692 (16)	0.6204 (3)	0.17395 (15)	0.0483 (6)	
C1	0.05293 (19)	0.1916 (4)	0.09291 (18)	0.0315 (6)	
C2	0.0772 (2)	0.0219 (4)	0.1048 (2)	0.0404 (8)	
H2	0.0502	−0.0489	0.1378	0.049*	
C3	0.1409 (2)	−0.0426 (5)	0.0681 (2)	0.0440 (8)	
H3	0.1583	−0.1580	0.0767	0.053*	
C4	0.1797 (2)	0.0591 (5)	0.0188 (2)	0.0405 (8)	
C5	0.1524 (2)	0.2261 (5)	0.00523 (19)	0.0398 (8)	
H5	0.1773	0.2952	−0.0300	0.048*	
C6	0.0891 (2)	0.2942 (4)	0.04216 (18)	0.0374 (7)	
H6	0.0710	0.4091	0.0328	0.045*	
C7	0.2519 (2)	−0.0112 (6)	−0.0184 (2)	0.0555 (10)	
H7A	0.2885	−0.0908	0.0205	0.083*	
H7B	0.2889	0.0820	−0.0297	0.083*	
H7C	0.2250	−0.0705	−0.0700	0.083*	
C8	−0.0103 (2)	0.6568 (4)	0.27908 (19)	0.0354 (7)	
C9	−0.0739 (2)	0.7409 (4)	0.2199 (2)	0.0431 (8)	
H9	−0.0693	0.7457	0.1642	0.052*	
C10	−0.1442 (2)	0.8180 (5)	0.2433 (2)	0.0470 (9)	
H10	−0.1882	0.8749	0.2028	0.056*	
C11	−0.1520 (2)	0.8138 (4)	0.3245 (2)	0.0436 (8)	
C12	−0.0872 (2)	0.7298 (5)	0.3823 (2)	0.0484 (9)	
H12	−0.0918	0.7247	0.4380	0.058*	
C13	−0.0163 (2)	0.6537 (5)	0.3610 (2)	0.0423 (8)	
H13	0.0283	0.5994	0.4019	0.051*	
C14	−0.2289 (3)	0.8997 (6)	0.3480 (3)	0.0650 (12)	
H14A	−0.2802	0.8236	0.3364	0.098*	
H14B	−0.2438	1.0043	0.3158	0.098*	
H14C	−0.2128	0.9273	0.4070	0.098*	
C15	0.0831 (2)	0.2258 (4)	0.29713 (19)	0.0337 (7)	
C16	0.0375 (2)	0.1710 (4)	0.35489 (19)	0.0369 (7)	
C17	0.0761 (2)	0.0474 (5)	0.4111 (2)	0.0442 (8)	
H17	0.0463	0.0097	0.4513	0.053*	
C18	0.1572 (2)	−0.0216 (5)	0.4098 (2)	0.0494 (9)	
H18	0.1821	−0.1084	0.4478	0.059*	
C19	0.2022 (2)	0.0359 (5)	0.3529 (2)	0.0493 (9)	
H19	0.2586	−0.0100	0.3526	0.059*	
C20	0.1653 (2)	0.1601 (5)	0.2965 (2)	0.0407 (8)	
H20	0.1962	0.1999	0.2576	0.049*	
C21	−0.0503 (2)	0.2395 (4)	0.3584 (2)	0.0414 (8)	
H21	−0.0813	0.3076	0.3138	0.050*	
O5	−0.08438 (19)	0.2136 (4)	0.41477 (18)	0.0640 (8)	

### Atomic displacement parameters (Å^2^)

**Table:**

	*U*^11^	*U*^22^	*U*^33^	*U*^12^	*U*^13^	*U*^23^
N1	0.0402 (14)	0.0320 (14)	0.0352 (14)	0.0043 (12)	0.0114 (11)	0.0042 (11)
S1	0.0333 (4)	0.0341 (4)	0.0357 (4)	0.0005 (3)	0.0072 (3)	0.0069 (3)
O1	0.0383 (12)	0.0377 (13)	0.0486 (13)	−0.0074 (10)	0.0113 (10)	0.0068 (11)
O2	0.0415 (12)	0.0433 (14)	0.0422 (13)	0.0095 (11)	0.0075 (10)	0.0120 (11)
S2	0.0344 (4)	0.0342 (4)	0.0414 (5)	−0.0026 (3)	0.0103 (3)	0.0029 (3)
O3	0.0388 (13)	0.0427 (14)	0.0571 (15)	−0.0027 (11)	0.0020 (11)	−0.0049 (12)
O4	0.0556 (15)	0.0426 (14)	0.0538 (15)	−0.0018 (12)	0.0270 (12)	0.0103 (12)
C1	0.0266 (14)	0.0334 (16)	0.0325 (15)	−0.0025 (13)	0.0034 (12)	0.0049 (13)
C2	0.0460 (19)	0.0364 (18)	0.0383 (17)	−0.0005 (15)	0.0089 (15)	0.0067 (14)
C3	0.051 (2)	0.0367 (19)	0.0428 (19)	0.0053 (16)	0.0086 (16)	−0.0017 (15)
C4	0.0357 (16)	0.051 (2)	0.0325 (16)	−0.0022 (16)	0.0042 (13)	−0.0082 (15)
C5	0.0375 (17)	0.050 (2)	0.0300 (16)	−0.0093 (16)	0.0050 (13)	−0.0003 (15)
C6	0.0398 (17)	0.0386 (18)	0.0321 (16)	−0.0044 (15)	0.0054 (13)	0.0042 (14)
C7	0.048 (2)	0.072 (3)	0.049 (2)	0.000 (2)	0.0151 (17)	−0.013 (2)
C8	0.0385 (17)	0.0307 (16)	0.0381 (17)	−0.0076 (14)	0.0113 (13)	0.0012 (13)
C9	0.0480 (19)	0.0396 (19)	0.0425 (19)	−0.0022 (16)	0.0121 (16)	0.0070 (15)
C10	0.0388 (18)	0.0388 (19)	0.062 (2)	0.0080 (16)	0.0084 (17)	0.0078 (17)
C11	0.0380 (18)	0.0352 (18)	0.061 (2)	−0.0084 (15)	0.0192 (16)	−0.0015 (16)
C12	0.052 (2)	0.050 (2)	0.048 (2)	−0.0018 (18)	0.0218 (17)	−0.0031 (17)
C13	0.0465 (19)	0.0402 (19)	0.0387 (18)	0.0026 (16)	0.0074 (15)	−0.0009 (15)
C14	0.051 (2)	0.059 (3)	0.094 (3)	0.007 (2)	0.034 (2)	0.000 (2)
C15	0.0343 (15)	0.0332 (17)	0.0322 (15)	0.0021 (13)	0.0052 (12)	0.0026 (13)
C16	0.0391 (17)	0.0383 (18)	0.0340 (16)	−0.0036 (14)	0.0105 (13)	0.0015 (14)
C17	0.053 (2)	0.046 (2)	0.0355 (17)	−0.0029 (17)	0.0144 (16)	0.0072 (15)
C18	0.053 (2)	0.045 (2)	0.046 (2)	0.0087 (18)	0.0053 (17)	0.0113 (17)
C19	0.0405 (18)	0.051 (2)	0.055 (2)	0.0133 (17)	0.0096 (16)	0.0113 (18)
C20	0.0330 (16)	0.045 (2)	0.0456 (19)	0.0006 (15)	0.0122 (14)	0.0056 (15)
C21	0.0400 (17)	0.0384 (19)	0.049 (2)	−0.0024 (15)	0.0170 (16)	0.0027 (15)
O5	0.0688 (18)	0.0678 (19)	0.0707 (18)	0.0055 (15)	0.0466 (16)	0.0109 (15)

### Geometric parameters (Å, º)

**Table:**

N1—C15	1.452 (4)	C9—C10	1.387 (5)
N1—S2	1.691 (3)	C9—H9	0.9500
N1—S1	1.697 (3)	C10—C11	1.390 (5)
S1—O2	1.422 (2)	C10—H10	0.9500
S1—O1	1.423 (2)	C11—C12	1.384 (5)
S1—C1	1.745 (3)	C11—C14	1.507 (5)
S2—O4	1.416 (2)	C12—C13	1.375 (5)
S2—O3	1.423 (2)	C12—H12	0.9500
S2—C8	1.746 (3)	C13—H13	0.9500
C1—C6	1.385 (4)	C14—H14A	0.9800
C1—C2	1.391 (5)	C14—H14B	0.9800
C2—C3	1.380 (5)	C14—H14C	0.9800
C2—H2	0.9500	C15—C20	1.381 (4)
C3—C4	1.387 (5)	C15—C16	1.396 (4)
C3—H3	0.9500	C16—C17	1.385 (5)
C4—C5	1.385 (5)	C16—C21	1.483 (5)
C4—C7	1.512 (5)	C17—C18	1.378 (5)
C5—C6	1.388 (5)	C17—H17	0.9500
C5—H5	0.9500	C18—C19	1.384 (5)
C6—H6	0.9500	C18—H18	0.9500
C7—H7A	0.9800	C19—C20	1.382 (5)
C7—H7B	0.9800	C19—H19	0.9500
C7—H7C	0.9800	C20—H20	0.9500
C8—C9	1.386 (5)	C21—O5	1.203 (4)
C8—C13	1.393 (4)	C21—H21	0.9500
			
C15—N1—S2	118.6 (2)	C8—C9—C10	119.0 (3)
C15—N1—S1	116.9 (2)	C8—C9—H9	120.5
S2—N1—S1	123.77 (16)	C10—C9—H9	120.5
O2—S1—O1	120.28 (14)	C9—C10—C11	121.5 (3)
O2—S1—N1	106.30 (14)	C9—C10—H10	119.2
O1—S1—N1	104.66 (13)	C11—C10—H10	119.2
O2—S1—C1	110.77 (14)	C12—C11—C10	118.2 (3)
O1—S1—C1	108.91 (15)	C12—C11—C14	121.6 (4)
N1—S1—C1	104.58 (13)	C10—C11—C14	120.2 (4)
O4—S2—O3	120.32 (16)	C13—C12—C11	121.5 (3)
O4—S2—N1	107.97 (15)	C13—C12—H12	119.3
O3—S2—N1	104.50 (14)	C11—C12—H12	119.3
O4—S2—C8	109.75 (15)	C12—C13—C8	119.6 (3)
O3—S2—C8	108.96 (15)	C12—C13—H13	120.2
N1—S2—C8	104.02 (14)	C8—C13—H13	120.2
C6—C1—C2	120.8 (3)	C11—C14—H14A	109.5
C6—C1—S1	119.9 (3)	C11—C14—H14B	109.5
C2—C1—S1	119.3 (2)	H14A—C14—H14B	109.5
C3—C2—C1	119.4 (3)	C11—C14—H14C	109.5
C3—C2—H2	120.3	H14A—C14—H14C	109.5
C1—C2—H2	120.3	H14B—C14—H14C	109.5
C2—C3—C4	120.7 (3)	C20—C15—C16	120.9 (3)
C2—C3—H3	119.6	C20—C15—N1	118.9 (3)
C4—C3—H3	119.6	C16—C15—N1	120.2 (3)
C5—C4—C3	119.1 (3)	C17—C16—C15	118.4 (3)
C5—C4—C7	120.7 (3)	C17—C16—C21	118.9 (3)
C3—C4—C7	120.2 (3)	C15—C16—C21	122.7 (3)
C4—C5—C6	121.2 (3)	C18—C17—C16	121.0 (3)
C4—C5—H5	119.4	C18—C17—H17	119.5
C6—C5—H5	119.4	C16—C17—H17	119.5
C1—C6—C5	118.8 (3)	C17—C18—C19	119.9 (3)
C1—C6—H6	120.6	C17—C18—H18	120.1
C5—C6—H6	120.6	C19—C18—H18	120.1
C4—C7—H7A	109.5	C20—C19—C18	120.2 (3)
C4—C7—H7B	109.5	C20—C19—H19	119.9
H7A—C7—H7B	109.5	C18—C19—H19	119.9
C4—C7—H7C	109.5	C15—C20—C19	119.6 (3)
H7A—C7—H7C	109.5	C15—C20—H20	120.2
H7B—C7—H7C	109.5	C19—C20—H20	120.2
C9—C8—C13	120.1 (3)	O5—C21—C16	123.5 (3)
C9—C8—S2	119.7 (2)	O5—C21—H21	118.2
C13—C8—S2	120.2 (3)	C16—C21—H21	118.2
			
C15—N1—S1—O2	168.6 (2)	O4—S2—C8—C13	−160.5 (3)
S2—N1—S1—O2	−21.4 (2)	O3—S2—C8—C13	−26.8 (3)
C15—N1—S1—O1	40.3 (2)	N1—S2—C8—C13	84.2 (3)
S2—N1—S1—O1	−149.72 (18)	C13—C8—C9—C10	−1.5 (5)
C15—N1—S1—C1	−74.1 (2)	S2—C8—C9—C10	178.6 (3)
S2—N1—S1—C1	95.8 (2)	C8—C9—C10—C11	0.5 (5)
C15—N1—S2—O4	138.1 (2)	C9—C10—C11—C12	−0.1 (5)
S1—N1—S2—O4	−31.7 (2)	C9—C10—C11—C14	179.6 (4)
C15—N1—S2—O3	8.9 (3)	C10—C11—C12—C13	0.7 (5)
S1—N1—S2—O3	−160.91 (18)	C14—C11—C12—C13	−179.0 (4)
C15—N1—S2—C8	−105.4 (2)	C11—C12—C13—C8	−1.7 (6)
S1—N1—S2—C8	84.9 (2)	C9—C8—C13—C12	2.1 (5)
O2—S1—C1—C6	26.1 (3)	S2—C8—C13—C12	−178.0 (3)
O1—S1—C1—C6	160.6 (2)	S2—N1—C15—C20	−80.4 (3)
N1—S1—C1—C6	−88.0 (3)	S1—N1—C15—C20	90.1 (3)
O2—S1—C1—C2	−155.9 (2)	S2—N1—C15—C16	100.8 (3)
O1—S1—C1—C2	−21.5 (3)	S1—N1—C15—C16	−88.7 (3)
N1—S1—C1—C2	89.9 (3)	C20—C15—C16—C17	−0.7 (5)
C6—C1—C2—C3	2.7 (5)	N1—C15—C16—C17	178.0 (3)
S1—C1—C2—C3	−175.2 (3)	C20—C15—C16—C21	179.2 (3)
C1—C2—C3—C4	−1.0 (5)	N1—C15—C16—C21	−2.0 (5)
C2—C3—C4—C5	−1.4 (5)	C15—C16—C17—C18	−0.8 (5)
C2—C3—C4—C7	177.6 (3)	C21—C16—C17—C18	179.3 (3)
C3—C4—C5—C6	2.1 (5)	C16—C17—C18—C19	1.7 (6)
C7—C4—C5—C6	−177.0 (3)	C17—C18—C19—C20	−1.2 (6)
C2—C1—C6—C5	−2.0 (5)	C16—C15—C20—C19	1.2 (5)
S1—C1—C6—C5	175.9 (2)	N1—C15—C20—C19	−177.5 (3)
C4—C5—C6—C1	−0.4 (5)	C18—C19—C20—C15	−0.3 (6)
O4—S2—C8—C9	19.4 (3)	C17—C16—C21—O5	11.7 (5)
O3—S2—C8—C9	153.1 (3)	C15—C16—C21—O5	−168.3 (4)
N1—S2—C8—C9	−95.9 (3)		

### Hydrogen-bond geometry (Å, º)

**Table:**

*D*—H···*A*	*D*—H	H···*A*	*D*···*A*	*D*—H···*A*
C6—H6···O2^i^	0.95	2.62	3.261 (4)	125

Symmetry code: (i) −*x*, −*y*+1, −*z*.
